# Successful Salvage of a Ruptured Testis Following Severe Blunt Trauma in a Polytrauma Patient

**DOI:** 10.7759/cureus.87265

**Published:** 2025-07-04

**Authors:** Athanasios Zachariou, Aris Kaltsas, Grigorios Daligaros, Ioannis Giannakis, Nikolaos Sofikitis

**Affiliations:** 1 Urology Department, University of Ioannina, Ioannina, GRC; 2 Third Department of Urology, Attikon University Hospital, School of Medicine, National and Kapodistrian University of Athens, Athens, GRC

**Keywords:** emergency surgery, hematoma, pelvic fracture, scrotal trauma, testicular function

## Abstract

Testicular rupture is a rare but serious complication of blunt scrotal trauma, often linked to high-impact injuries. We report the case of a 20-year-old male with pelvic fractures and a high-riding right testis following a motorcycle accident. Imaging confirmed left testicular rupture with tunica albuginea disruption and hematoma. Surgery was performed eight hours post-injury, involving tunica repair and debridement. Follow-up ultrasound showed preserved arterial flow despite reduced testicular size. This case underscores the need for prompt urological assessment in blunt trauma cases, ensuring timely intervention to maximize testicular salvage and function.

## Introduction

Scrotal and testicular trauma represent an uncommon category of injuries, accounting for significantly less than 1% of all trauma cases in the United States [1]. Their low incidence has been linked to the scrotum’s mobility, which often helps it avoid significant direct impact [2]. However, in cases involving high-energy blunt trauma, such as those sustained in motorcycle accidents, damage to the scrotal sac and testis can be severe. Testicular rupture has been reported in nearly 50% of such incidents [3]. According to data from the National Trauma Data Bank, the prevalence of scrotal and testicular trauma is only around 0.23%, underscoring just how infrequently these injuries are seen [1]. Despite their rarity, these injuries carry the risk of serious consequences if not promptly recognized and treated. Delayed diagnosis or misjudgment of injury severity may lead to chronic scrotal pain, infertility, infection, or the necessity for orchiectomy [4]. As such, timely surgical assessment and intervention are essential to preserving testicular function and reducing long-term complications. This report presents a case of blunt scrotal trauma resulting in testicular rupture, illustrating the diagnostic challenges involved and emphasizing the importance of immediate surgical management.

## Case presentation

A 20-year-old male presented to the emergency department following a high-impact motorcycle accident, reporting severe abdominal and bilateral hip pain. He was assigned a red triage category.

On initial evaluation, the patient’s blood pressure was 120/80 mmHg, heart rate 89 beats per minute, respiratory rate 18 breaths per minute, temperature 36.8°C, and oxygen saturation 98% on room air. Hematocrit was 32%. The trauma protocol was activated for urgent evaluation and treatment. The patient had no significant medical or surgical history. Physical examination revealed bilateral hip instability and marked pelvic tenderness. Plain radiographs and computed tomography (CT) scans confirmed multiple comminuted pelvic fractures, prompting the application of a pelvic binder for stabilization. During the primary survey, there were no signs of lower urinary tract injury. No blood was noted at the urethral meatus, and the digital rectal examination was unremarkable. He was subsequently taken to the operating room for urgent orthopedic management.

**Figure 1:**
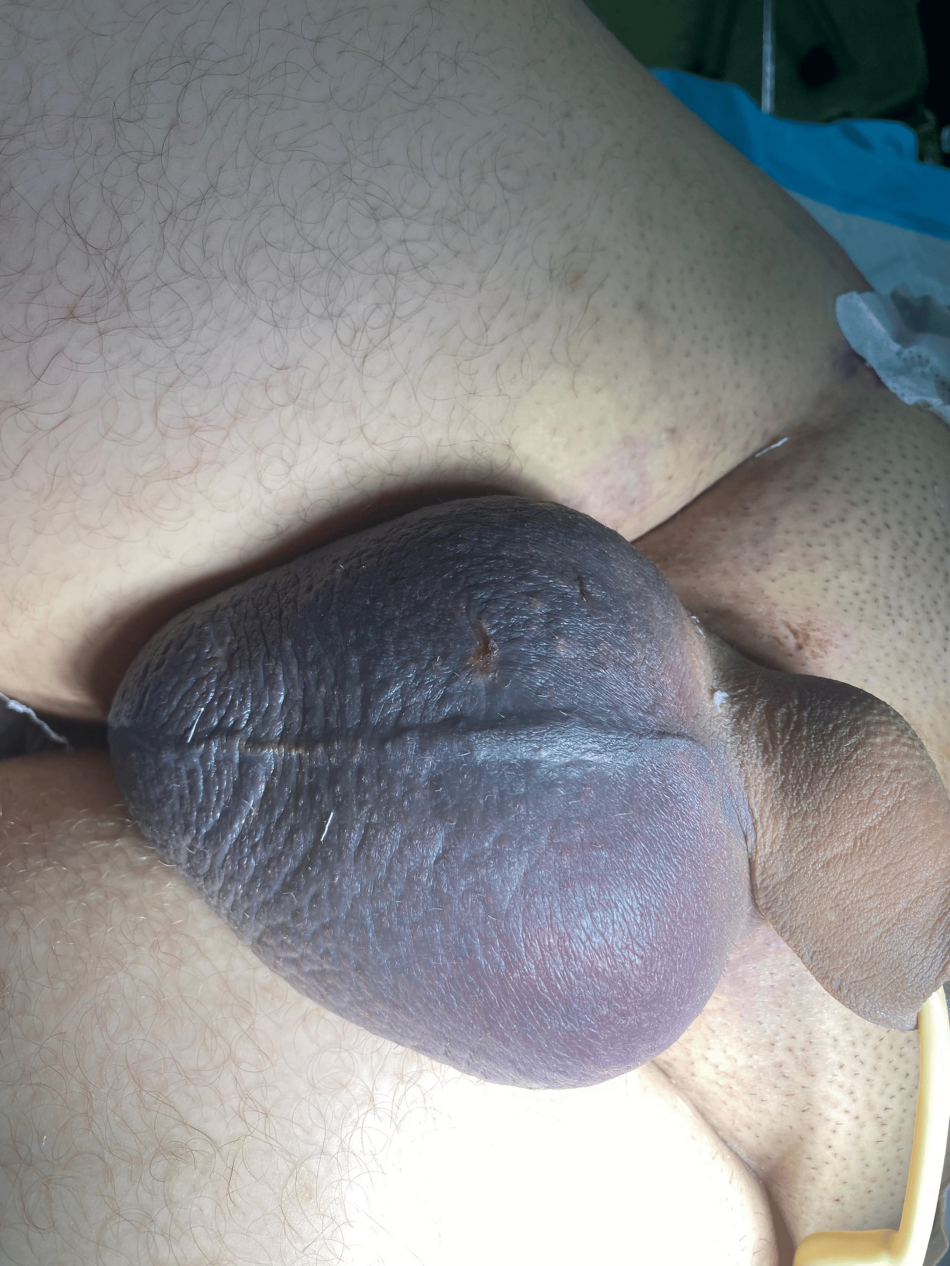
Initial presentation with diffuse scrotal discoloration The figure is presented with the patient’s informed consent.

During the secondary survey, scrotal discoloration was observed, along with a high-riding right testis noted in the right inguinal canal (Figure 1). The discoloration progressively worsened over time. A scrotal ultrasound demonstrated disruption of the left tunica albuginea, an eccentric echogenicity within the left testicular parenchyma, and a heterogeneous, avascular collection consistent with hematoma. The right testis appeared normal, exhibiting a homogeneous echotexture without evidence of rupture. These findings were indicative of left testicular rupture, diagnosed approximately eight hours after the initial trauma.

**Figure 2:**
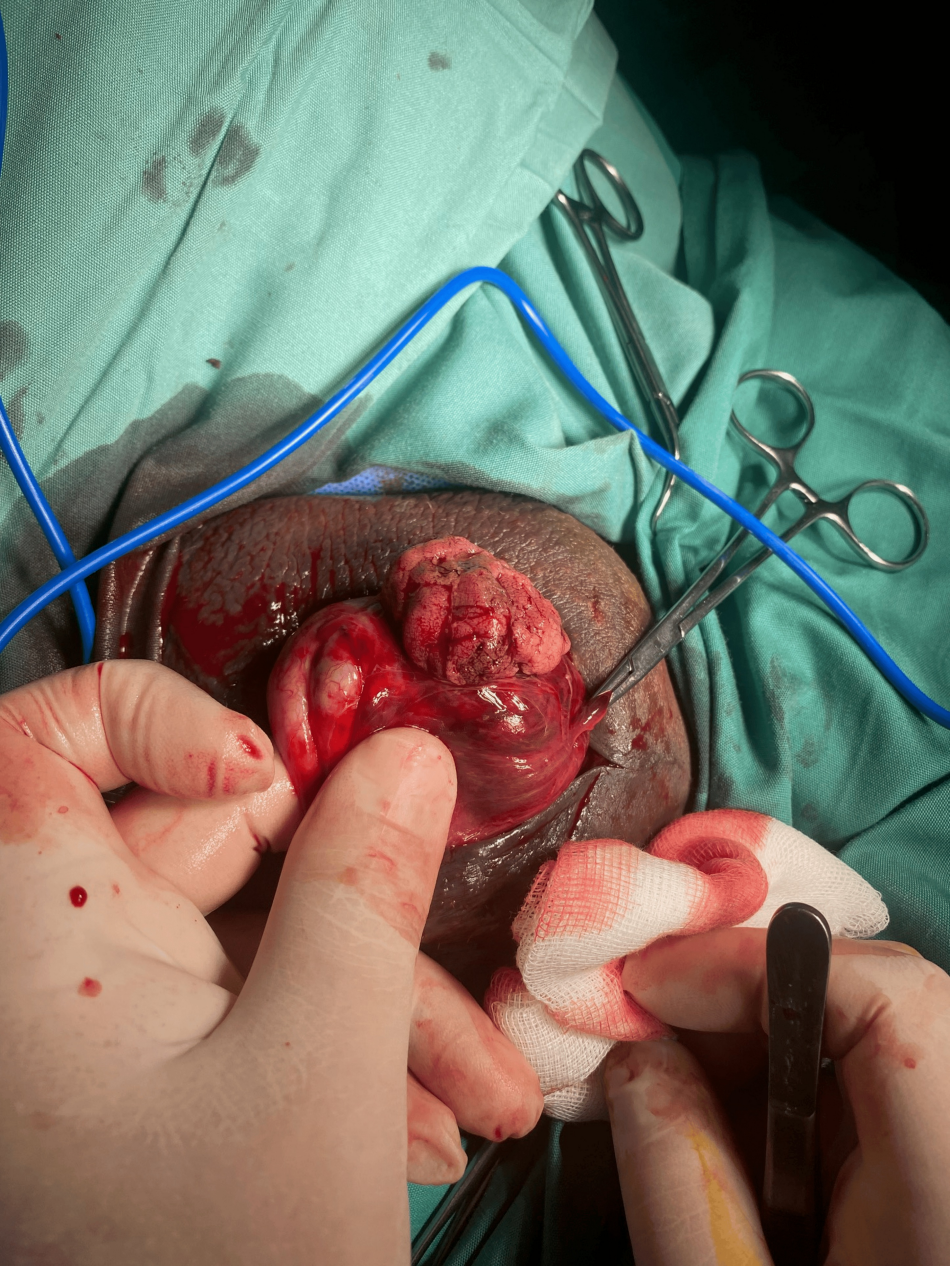
Traumatic rupture of the left testis with extrusion of testicular parenchyma through the tunica albuginea The figure is presented with the patient’s informed consent.

Given the strong suspicion of testicular rupture, an urgent surgical exploration was performed. Intraoperatively, the necrotic portion of the left testicular parenchyma was excised, and the tunica albuginea was repaired with interrupted absorbable sutures. A sizeable hematoma was evacuated, and meticulous hemostasis was achieved (Figure 2). The right testis, which showed no abnormalities on ultrasound, was not surgically explored.

**Figure 3:**
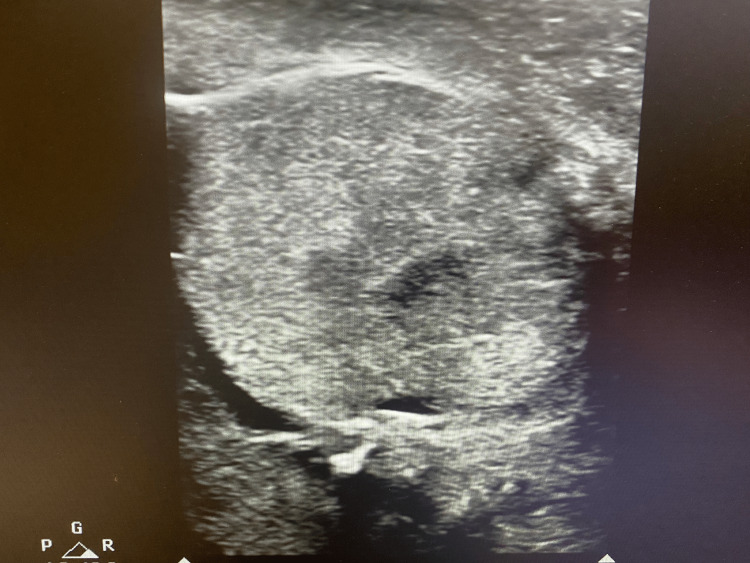
Ultrasound of the left testis revealing a decrease in the size The figure is presented with the patient’s informed consent.

**Figure 4:**
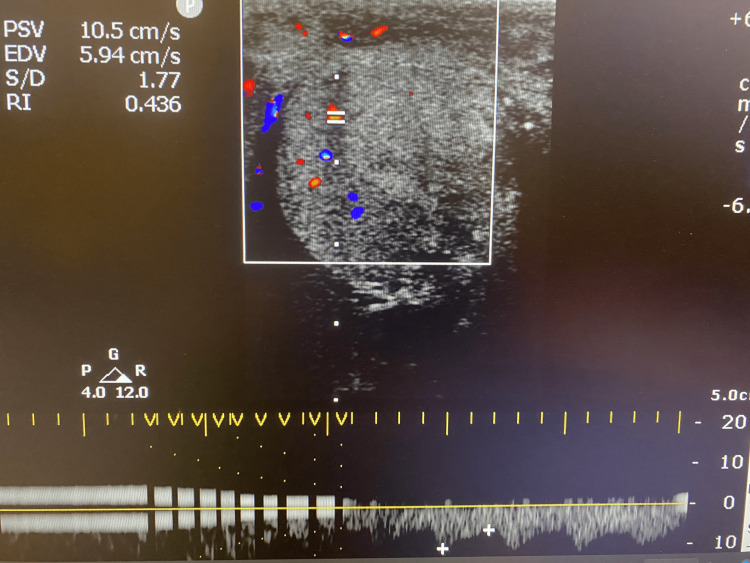
Doppler ultrasound of the left testis revealing preservation of arterial blood flow The figure is presented with the patient’s informed consent.

On postoperative day 1, a Doppler ultrasound revealed a decrease in the size of the left testis but preservation of arterial blood flow. Despite initial concerns about the potential for complete loss of function, the patient’s long-term prognosis remained favorable (Figures 3, 4). At follow-up, he reported no significant complications, including chronic pain or infection, and continued imaging studies confirm sustained perfusion to the left testis. Hormonal evaluations at three and six months post-injury were unremarkable. A semen analysis performed six months after the testicular injury showed normal values, with a sperm concentration of 16 million/mL, and normal motility, vitality, and morphology. However, there were no available pre-injury semen analysis data for comparison. Further assessments are planned to evaluate fertility and endocrine function over time, although current findings suggest a positive outlook for testicular viability.

## Discussion

Blunt testicular trauma is an underrecognized yet significant clinical issue, frequently eclipsed by more life-threatening injuries in polytrauma settings [5]. Despite constituting a small proportion of overall trauma admissions, testicular injuries can lead to profound long-term sequelae, including chronic pain, infertility, and considerable psychological distress [2]. In this case, the patient sustained multiple high-energy orthopedic injuries that required urgent attention. Although high-impact trauma mandates a complete primary survey according to Advanced Trauma Life Support (ATLS) guidelines, including a genital examination, testicular injuries can still be overlooked in these complex settings.

Early identification of testicular rupture is essential given that timely surgical exploration directly correlates with improved rates of testicular salvage [6]. Although diagnostic imaging plays a central role, certain clinical features warrant high suspicion. For instance, scrotal discoloration, significant swelling, or a “high-riding” testis - especially in the context of blunt impact - should prompt immediate ultrasonographic evaluation. Doppler ultrasound is widely regarded as the first-line imaging modality owing to its ability to rapidly evaluate testicular parenchyma, detect disruptions of the tunica albuginea, and assess blood flow [7]. Nevertheless, extensive hematoma or regional edema can diminish image clarity and lead to underestimation of the severity of the injury. When ultrasonographic findings are ambiguous and clinical suspicion remains elevated, surgical exploration is advised to avoid delayed intervention and potential adverse outcomes [8].

The timing of operative repair holds critical importance for preserving testicular tissue viability. Research consistently illustrates that exploring and repairing testicular injuries within six hours of the traumatic event substantially increases the likelihood of retaining functional testicular tissue: in torsion cases, salvage rates reach 80-100 % when treated within 6 hours, and in blunt ruptures, operating within 72 hours yields up to 90 % salvage [9]. Conversely, delays in surgical management facilitate ischemic changes, progressive necrosis, and heightened risk for postoperative complications. In the case described, although surgery was performed approximately eight hours after the initial injury, partial salvage of the injured testis was still achieved, underscoring that even delayed intervention can mitigate some of the detrimental consequences of testicular rupture. The intraoperative steps of debriding nonviable tissue, suturing the disrupted tunica albuginea, and evacuating the hematoma are critical to halting ongoing ischemic damage, reducing infection risk, and optimizing remaining testicular function [10].

Long-term follow-up is paramount for assessing both endocrine integrity and fertility potential. Even when salvage is successful, patients may still encounter decreased spermatogenesis, reduced testosterone production, or formation of anti-sperm antibodies that can impair reproductive outcomes. Periodic evaluation, including hormonal profiling and semen analysis, is recommended to identify potential deficits that may necessitate pharmacological intervention or assisted reproductive technologies [11]. Additionally, psychological support should be integrated into the care plan, given the potential for self-image concerns, anxiety, or depressive symptoms associated with genital trauma and possible partial or complete loss of a testis. Addressing these challenges through counseling, discussing the option of testicular prosthesis placement, and offering referral to mental health services can significantly enhance a patient’s overall well-being [12].

While surgical repair remains the primary treatment, adjunctive pharmacotherapy, including hormonal therapy with human chorionic gonadotropin (hCG) or selective estrogen receptor modulators (SERMs), may be considered in cases of hypogonadism following testicular trauma to support the restoration of endocrine and spermatogenic function, although evidence in the trauma setting remains limited [13].

## Conclusions

This case underscores the importance of a comprehensive trauma evaluation. While orthopedic and hemodynamic stabilization may be the initial priorities, concurrent assessment for scrotal and testicular injury should not be overlooked. Testicular rupture should always be included in the differential diagnosis when evaluating a polytrauma patient with significant lower abdominal or pelvic injuries. Timely scrotal ultrasound and surgical exploration are crucial for optimizing outcomes and testicular salvage.
